# Fear of Fishers: Human Predation Explains Behavioral Changes in Coral Reef Fishes

**DOI:** 10.1371/journal.pone.0022761

**Published:** 2011-08-10

**Authors:** Fraser A. Januchowski-Hartley, Nicholas A. J. Graham, David A. Feary, Tau Morove, Joshua E. Cinner

**Affiliations:** 1 Australian Research Council Centre of Excellence for Coral Reef Studies, James Cook University, Townsville, Queensland, Australia; 2 School of the Environment, University of Technology, Sydney, New South Wales, Australia; 3 Wildlife Conservation Society, Papua New Guinea Marine Program, Kavieng, New Ireland Province, Papua New Guinea; University of Western Australia, Zimbabwe

## Abstract

Prey flight decisions in response to predation risk are increasingly being considered in conservation and management decisions in the terrestrial realm, but are rarely considered in marine systems. This field-based study investigated how the behavioral response of coral reef fish families varied along a gradient of subsistence fishing pressure in Papua New Guinea. Specifically, we examined how fishing pressure was related to pre-flight behavior and flight initiation distance (FID), and whether FID was influenced by body size (centimeters total length), group size (including both con- and hetero-specific individuals), or life-history phase. Fishing pressure was positively associated with higher FID, but only in families that were primarily targeted by spear guns. Among these families, there were variable responses in FID; some families showed increased FID monotonically with fishing pressure, while others showed increased FID only at the highest levels of fishing pressure. Body size was more significant in varying FID at higher levels of fishing pressure. Although family-level differences in pre-flight behavior were reported, such behavior showed low concordance with fishing pressure. FID shows promise as a tool by which compliance and effectiveness of management of reef fisheries can be assessed.

## Introduction

Appropriate response to predation risk is one of the most important factors in enhancing fitness and reproductive success among animals [1], [2]. The most commonly used metric to assess prey decision making and wariness in the light of predation is flight initiation distance (FID) – the distance to which a predator can approach prey before the prey animal flees [3]. Research using this metric has given rise to an extensive theoretical framework, culminating in the theory of optimal FID, which states that “a prey animal will flee at the stage of an encounter at which maximal fitness is achieved” [2]. There are a range of factors that may influence when a prey animal makes the decision to flee from a predator [4]. These include: environmental factors (e.g., food patch quality [5]); refuge availability [6]; prey effects (e.g., previous experience of predation [7]); morphological defenses [8]; social defenses; and transmission of information through the prey population [9].

Increased wariness of prey species in the context of higher predation has been reported for both natural [10], [11] and human predation [12], [13]. Although this understanding of FID in predator/prey relationships has improved our ability to manage terrestrial animal populations, for example through the use of setback and buffer zones to minimize disturbance [14], there is a paucity of research on the impacts of human predation on the FID of marine species. Studies to date consistently show that fishing activity does influence FID. For example, coral reef fishes normally targeted by spear fishers showed lower FID within areas protected from fishing pressure [12], [15], while in New Caledonia, detection distance (mean distance from the transect line at which fishes were observed) was found to increase with intensity of fishing [16]. In parallel, within a New Zealand marine reserve, it was found that “approach distance” (synonymous with FID) of targeted fishes increased with distance from the centre of the marine reserve [17]. Although previous studies agree that fishing intensity directly influences FID, the role of body size and group size is more contested. In Papua New Guinea, fishes' body size was found to be non-significant as an explanatory factor for FID [12], contrary to studies elsewhere [15], [16], which found that larger sized fishes exhibited greater FID/mean detection distance. In the terrestrial literature, increased group size tends to be accompanied by increased FID [4], [18] contrary to data available on fish, where increased group size has generally been found to be associated with lower FID [4], [19].

Prey species wariness to predators may also be expressed through behaviors other than flight [7], [20]. Fishes are well-equipped for social learning and transfer of information, and alarm signals are often communicated through visual and other sensory systems [21]. Visually transmitted alarm signals can originate as a result of predator inspection behavior, where a prey fish fixates on a predator, and slowly swims towards it [22]. While inherently risky, this behavior may allow assessment of predator intent [23] and dissuasion of predation [24], while also advertising fitness to potential mates [25]. However, this behavior may make fishes particularly vulnerable to spear fishers, because it brings the fish closer to the fisher, and highlights the fish as a target. Although there is little empirical data, at higher fishing pressures fishes' behavior prior to flight would theoretically be expected to show declines in occurrence of ‘less wary’ behaviors (e.g. inspection), with ‘more wary’ behaviors (e.g., immediate flight or movement towards a refuge), becoming more frequent.

Despite over a decade passing since the effects of differing human predation on coral reef fish behavior were first identified in the literature [16], the importance of human-induced fish behavior in structuring fish communities is rarely considered within the conservation and fisheries management literature [26], [27], [28]. Although levels of artisanal fishing can vary widely, even low levels of subsistence fishing have been associated with dramatic declines in fishery target species [29]. While underwater visual census (UVC) of abundance and catch survey data are often used to assess the success of management in small-scale subsistence fisheries, they are subject to high variance [30] or may not provide the information necessary to accurately assess and manage the ecosystem over short temporal scales [31]. Changes in the structure of fish communities due to altered management practices may occur over multi-year to decadal scales [32], [33]. However, behavioral responses to altered fishing practices may express themselves over much shorter temporal scales [34], and the assessment methods above do not lend themselves to identifying such temporally rapid changes within reef fish communities. If differences in FID or other behaviors are driven by changes in management or compliance, monitoring of behavior may prove to be a tool that can quickly and accurately identify and assess the results of such changes. This may be particularly useful in the assessment of compliance with no-take areas (NTAs) or gear bans in coral reef and similar fisheries.

This study aims to clarify whether predictions made by anti-predator escape theory are reproduced within coral reef fisheries, and ascertain how different factors influencing FID interact as fishing pressure increases. The relevant predictions made by FID theory are: 1) as the intrinsic risk of predation and lethality of encounters increase FID should likewise increase; and 2) as prey increase in size, FID should also increase. We hypothesized that as fishing pressure increases, fish targeted by fishers will show increased wariness, and that this will be reflected in increases in FID and the type and frequency of pre-flight behavior. To explore these hypotheses, we examined FID at four coastal communities in PNG along a gradient of fishing intensity.

## Materials and Methods

### Ethics

All research involving human participants was approved by the James Cook University Human Ethics Committee and was conducted within University guidelines. Permission was asked for and received verbally from fishers who were shadowed in order to create the standardized snorkel methodology. Prior to conducting research in all community tenure areas, verbal permission to access the protected or fished areas was sought from the local community, and where appropriate, clan leaders. All research was only conducted after permission had been granted. This study was conducted under Fraser Januchowski-Hartley's special exemption/researcher visa for Papua New Guinea, number 99902040235.

### Study sites

Flight initiation distance (FID) of coral reef fishes was assessed at four sites in the Tigak and Tsoi Islands of New Ireland Province, Papua New Guinea between July and September 2010. We surveyed three communities with varying levels of fishing pressure (Ungakum - low, Nusa - intermediate, and Mongol - high), and one community (Kavulik) who comply with a no-take fisheries closure (NTA) that has been in place since February 2008 (TM, personal observation). Previous research indicated that these areas were appropriate for this study because fishing activities primarily consist of spear gun and hand line, with fishers often using both gears within the same fishing trip (JE Cinner, unpublished data). Each of the communities have exclusive access rights to their fishing ground, with the exception of Mongol, which, as a community of migrants and located adjacent to the provincial capital of Kavieng, has seen adherence to customary tenure rights fade (FAJ, TM, personal observation).

To estimate fishing pressure within each community, we used the average number of reef-associated fishing trips per week per household (i.e., we removed gears that target pelagic fishes such as trolling, and gears commonly used in lagoons, such as nets) from previous studies that surveyed household fishing practices in these communities [35]. To account for population growth since the earlier surveys (2002 in Mongol and Nusa, 2009 in Kavulik and Ungakum) we re-counted the total number of households in each community in 2010. To calculate fishing ground size, the limits of fringing reef that were claimed as exclusive fishing grounds by each community were marked by GPS and linear reef distance estimated by digitally tracing the reef edge. We multiplied the average fishing trips per week by the total number of households and divided this by the length of each community's respective fishing ground to develop a measure of fishing trips per linear kilometer of reef per week for each community, and used a finite population correction factor to estimate the error associated with each estimate. The estimate of fishing pressure at Mongol obtained by this method is potentially lower than the actual fishing pressure, due to loss of tenure rights and fishing within the fishing ground by non-residents. Mongol's relative position as the site of highest fishing pressure means that any underestimates of fishing pressure at this site should not affect our interpretation of the results.

To allow comparisons of FID across all four communities, underwater surveys were conducted along approximately one linear kilometer of continuous fringing reef at each area. The majority of spear fishing in the region occurs between the crest and the 10 m depth contour on the reef slope, and all surveys were conducted in this reef zone. Within each area surveyed, benthic complexity was assessed visually using 8–10 replicate 50 m transects (to control for availability of potential refuge for fishes between areas). Each transect was given a benthic complexity score between 0 and 5 [36]: 0 = no vertical relief; 1 = low and sparse relief; 2 = low but widespread relief; 3 = moderately complex; 4 = very complex; and 5 = exceptionally complex. This method has been shown to be highly correlated with the linear versus contour complexity measure, reef height and abundance of holes 10–70 cm diameter when conducted by experienced observers [37], and captures the important characteristics of coral reef substrates as refuge.

### Selection of Focal Families

We selected focal families based on records of fishery catches by local communities in Kavieng [38] and other areas of PNG [39]. Focal families were also tractable to investigation (e.g., diurnally active, reef resident), and were present in sufficient abundance at the study areas to meet power requirements. Six families were chosen for this research: surgeonfish (F. Acanthuridae), triggerfish (F. Balistidae), snapper (F. Lutjanidae), goatfish (F. Mullidae), parrotfish (F. Scaridae) and grouper (F. Serranidae). Acanthuridae and Scaridae make up the majority of the spear gun catch in PNG, while the Balistidae, Lutjanidae and Serranidae are primarily caught by hook and line [38], [39]. Mullidae are caught by both gears at approximately the same relative frequency compared to other families [39]. In total FID was measured in 680 coral reef fishes that ranged in size from 10 to 50 centimeters total length (cm TL), encompassing 54 species across the six families.

### Behavior and Flight Initiation Distance

Although previous studies on FID of reef fishes have used SCUBA divers as predation stimuli [12], [15], [17], [20], all FID surveys within the present work were based on snorkeling as our interest was in how fishes respond to local spear fishers (who do not use SCUBA) [40]. All FID surveys were conducted by the primary author (FAJ).

To develop a standardized and repeatable method of approaching target fish that closely mimicked PNG spear fishing techniques, we consulted with local spear fishers in the region and observed them during fishing activities. Many spear fishers have idiosyncratic behaviors, and here we developed our method from similarities between spear fishers. This involved first identifying a target fish from the surface, prior to quietly (minimizing surface noise and air bubbles) descending to the benthos at approximately 8–10 m from the targeted fish. After descent, the observer lay motionless on the benthos between 10–20 seconds while re-orientating and ensuring the target fish had not been disturbed. The target fish was then approached at a steady swimming speed. When the fish started to flee a marker was dropped level with the head of the observer, and a second marker then placed at the location from which the fish fled. The distance (cm) between markers was then measured to obtain FID. The maximum FID obtained by Feary et al. [12] was approximately 8, consequently, in order to avoid beginning trials within FID of target fishes, all trials began outside this distance, and were conducted only when visibility was ≥10 m.

Fishes were only targeted for approach if they exhibited normal daily behavior (i.e., were not obviously alert to observer presence, fleeing from predators, or engaged in competition with con- or hetero-specifics). If line of sight between the target fish and observer was broken prior to flight, or if during the approach the target fish was chased by another fish, the trial was abandoned. Only fishes greater than 10 centimeters (cm TL) were approached as spear fishers will rarely target fishes under this size (FAJ, personal observation). For each fish, size (cm TL), behavior exhibited prior to flight (hereafter “pre-flight behavior”), group size, life-history phase (only for F. Scaridae) and refuge choice were recorded. Pre-flight behavior was assigned into five broad types of behavioral response, ranging from most-wary to least-wary behavior, based on perceived increase in vulnerability to fishers. These were: “none” – the fish fled without changing behavior; “tacking” – the fish halted activity and slowly swam away tacking from side to side before fleeing; “orientation” – the fish orientated to flee to a refuge; “watch” – the fish stopped current activity and turned towards the observer; and “inspect” – the fish moved towards the observer prior to flight.

To minimize the chance of approaching a target fish that had been disturbed by previous surveys, consecutive trials in the same area were conducted a minimum of 10 m apart. A pilot study found that after approximately 20 minutes of repeated FID surveys, most target fishes had vacated an area of approximately 30 linear meters of reef. Therefore, the observer moved steadily along the reef front during each sampling session, and did not revisit areas on consecutive days, in order to avoid both this response and habituation of fishes to his presence.

### Data Analyses

All data analyses were performed using MINITAB Version 14, with a significance level of *p*≤0.05. FID data was inspected for normality through quantile-quantile plots, while homogeneity of variance was determined using Levene's test. It was necessary to square root transform Acanthuridae FID data in order to meet assumptions of normality and homogeneity. To investigate FID for each family between areas, we used analysis of covariance (ANCOVA), with fish body size, group size and life-history stage (F. Scaridae only) as covariates in the model. Where differences in FID were significant, we used a post-hoc Tukey's test to identify where FID differed. Where fish size or group size was significant in the model, we analyzed the effect of these continuous variables across all areas and independently within each area, using linear regression. This was done in order to partition the effects of fishing pressure from either body size or group size. In addition, separate one-way analyses of variance (ANOVA) were conducted to investigate whether there were differences in substrate rugosity between survey areas. Lastly, pre-flight behavior and refuge choice were analyzed using Pearson's Chi-squared to test the hypothesis that fishes in more heavily fished areas would show more wary behavior when confronted with a spear fisher. For the purposes of analysis the “watch” and “inspect” behaviors were merged.

## Results

**Figure 1:**
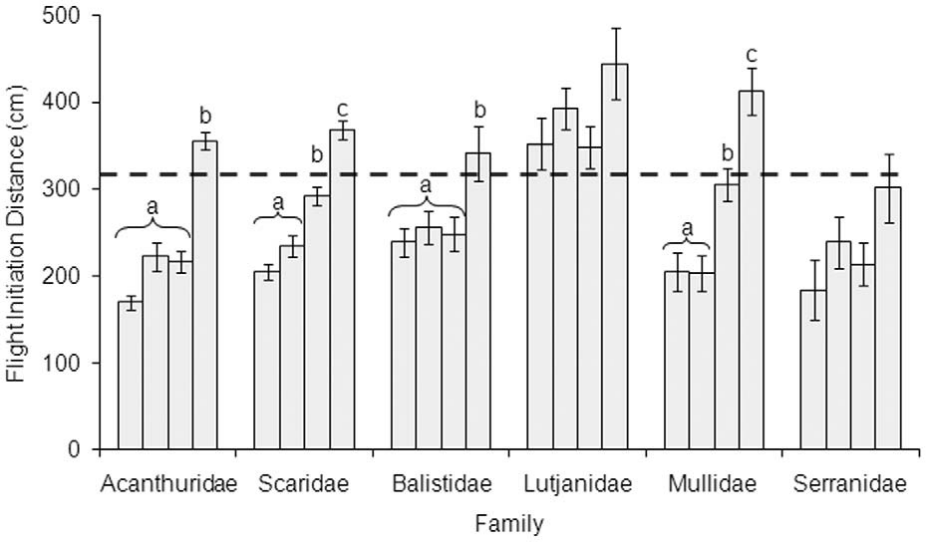
Mean flight initiation distance (FID) (cm ± S.E) at each fishing ground for six coral reef fish families. FID was estimated for individuals of six families of coral reef fishes at four different fishing pressures. From left to right within each family, bars are: Kavulik no-take area (no fishing); Ungakum (low fishing pressure); Nusa (intermediate fishing pressure); and Mongol (high fishing pressure). If significant differences existed in FID within families at different fishing grounds, grounds were grouped by similarity (a, b and c). Dashed line is maximum distance at which rifle-style spear guns used in New Ireland province are considered effective (approximately 310 cm, Feary et al. [12]).

Fishing pressure was highest at Mongol (147±38 trips/km/week), followed by Nusa (110±23 trips/km/week), then Ungakum (29±8 trips/km/week). FID increased with fishing pressure in the Acanthuridae, Scaridae, Balistidae and Mullidae (Table 1). Acanthuridae and Balistidae showed significant increases in FID at the highest fishing pressure (Mongol) when compared to all other areas (Fig. 1). Scaridae and Mullidae showed a steady trend of increasing FID, with low FID at unfished and lightly fished areas (Kavulik and Ungakum), moderate FID at intermediate fishing pressure (Nusa) and the highest FID at the highest fishing pressure (Mongol). FID did not significantly vary with fishing intensity for Lutjanidae or Serranidae. Overall, FID ranged from 27 to 722 cm. When compared to the maximum effective range of spear guns used in this region (310 cm) [12], only the Lutjanidae had a mean FID greater than spear gun range at all levels of fishing pressure, while Serranidae mean FID was never greater than spear gun range (Fig. 1). Only at the highest fishing pressure did other families show mean FID greater than maximum effective spear gun range (Fig. 1).

**Table 1:** Analysis of covariance (ANCOVA) results of flight initiation distance (cm) with fishing pressure as a fixed factor and fish body size (cm TL) and group size as co-variates.

Family (d.f.)	Factor	F	R^2^	P
Acanthuridae (3, 158)	fishing pressure	35.38	0.618	***^a^
	body size	43.88		***
	group size	10.94		**
Scaridae (3, 234)	fishing pressure	47.65	0.504	***
	body size	66.81		***
	group size	1.05		0.306
	life history stage	1.79		0.149
Balistidae (3, 56)	fishing pressure	5.26	0.357	**
	body size	22.51		**
	group size	0.04		0.845
Lutjanidae (3, 75)	fishing pressure	1.86	0.074	0.143
	body size	3.17		0.079
	group size	0.14		0.709
Mullidae (3, 76)	fishing pressure	18.08	0.487	***
	body size	10.70		**
	group size	2.01		0.160
Serranidae (3, 41)	fishing pressure	2.30	0.224	0.092
	body size	10.15		**
	group size	1.20		0.965

a *** = *p*<0.001; ** = *p*<0.01 and; * = *p*<0.05.

**Figure 2:**
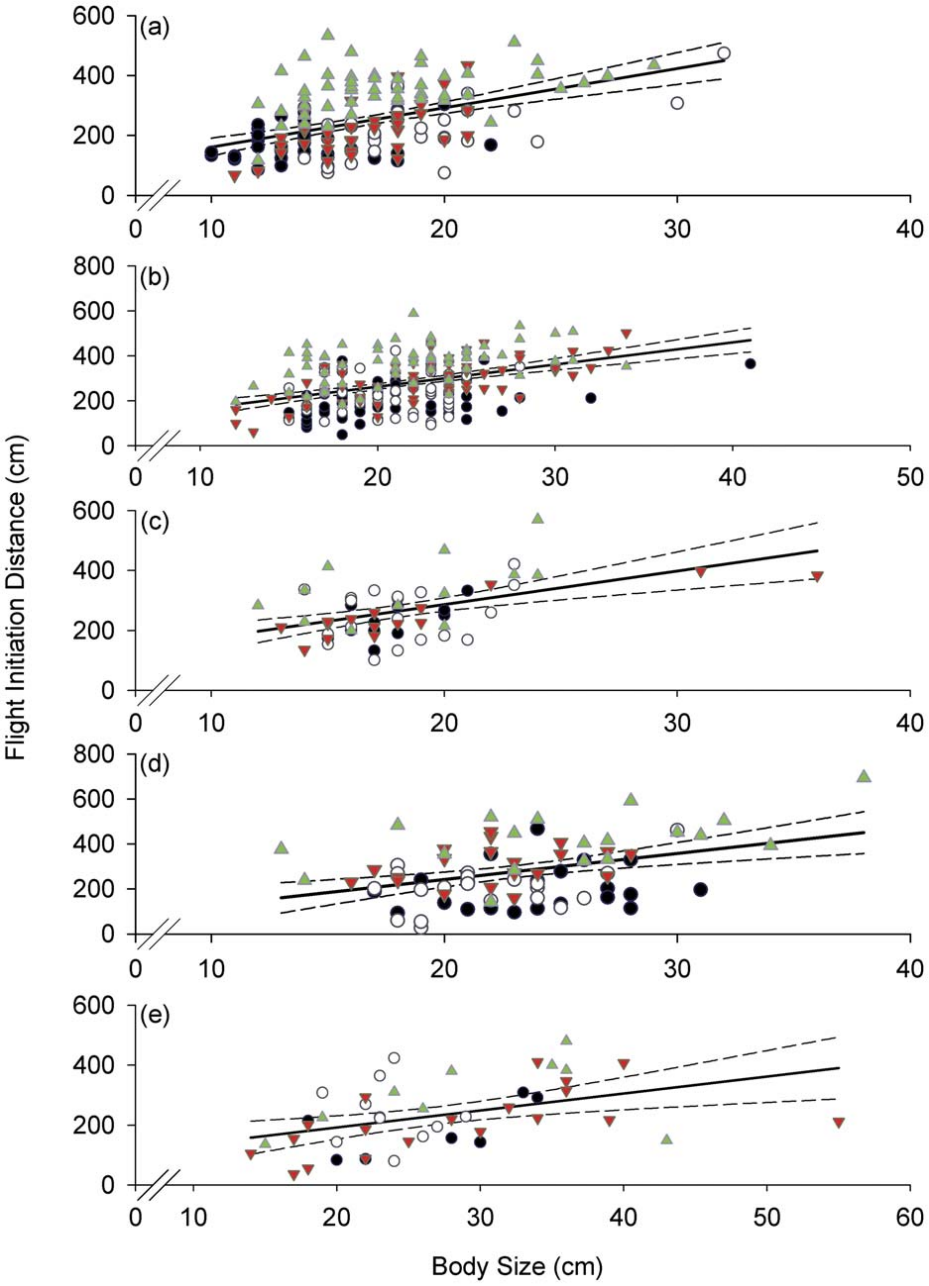
Effect of body size (cm TL) on flight initiation distance (cm). Flight initiation distance plotted against fish body size for: (a) Acanthuridae; (b) Scaridae; (c) Balistidae; (d) Mullidae; and (e) Serranidae. Black circles, open circles, inverted red triangles and upright green triangles represent Kavulik no-take area (no fishing), Ungakum (low fishing pressure), Nusa (intermediate fishing pressure) and Mongol (high fishing pressure) fishing grounds, respectively. Solid lines are significant linear regression across all grounds and dotted lines are 95% confidence intervals. For significance and R^2^ values see Table 2. Note that scales differ on both X and Y axes.

**Figure 3:**
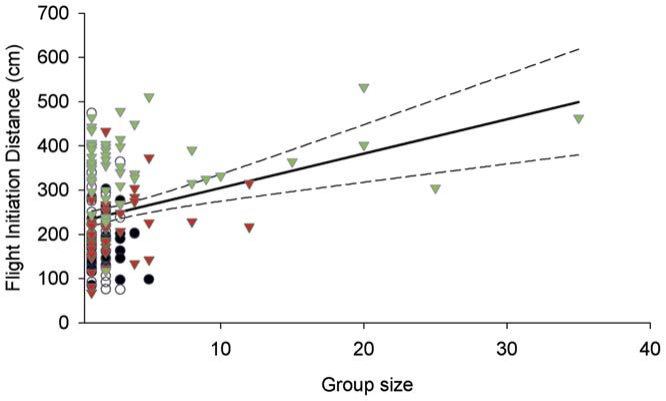
Effects of group size on flight initiation distance of Acanthuridae. Group size (number of individuals) plotted against FID (cm) for Acanthuridae. Black circles, open circles, red triangles and green triangles represent Kavulik no-take area (no fishing) Ungakum (low fishing pressure), Nusa (intermediate fishing pressure) and Mongol (high fishing pressure) fishing grounds respectively. Solid line is significant linear regression across all grounds and dotted lines are 95% confidence intervals. For significance and R^2^ values see Table 2.

All families except Lutjanidae showed a significant effect of fish body size on FID (Table 1). Linear regression analysis across all areas indicated that for all families, greater body size was predictive of greater FID (Fig. 2). When linear regression analysis was conducted for each family partitioned by fishing area, there was no significant relationship between fish body size and FID for the majority of families surveyed in unfished and lightly fished areas (Table 2). The heavily spear-fished Acanthuridae and Scaridae, showed a significant relationship between body size and FID at higher fishing pressures, while the less heavily spear-fished families only showed a significant relationship with intermediate fishing pressure (Balistidae and Serranidae), and at the highest fishing pressure (Mullidae) (Table 2). Group size only had a significant effect on FID for Acanthuridae (Table 1). Linear regression analysis for group size and FID for Acanthuridae indicated a significant relationship for all areas combined (R^2^ = 0.091, F_(1, 162)_ = 17.28, p<0.001) (Fig. 3), but not within grounds (Table 2). There was no effect of life history stage on FID of Scaridae (Table 1).

**Table 2:** R^2^ values of linear regression analysis of flight initiation distance with body size (cm TL) and group size reported by family and fishing ground.

	*Kavulik*	*Ungakum*	*Nusa*	*Mongol*	*All*
*Body Size*					
Acanthuridae	0.055	0.296**^a^	0.347***	0.139**	0.216***
Scaridae	0.156**	0.038	0.435***	0.188***	0.179***
Balistidae	0.245	0.060	0.762***	0.295	0.243***
Mullidae	0.024	0.173	0.047	0.244*	0.155***
Serranidae	0.415	0.026	0.315**	0.151	0.190**
*Group Size*					
Acanthuridae	0.015	0.027	0.064	0.060	0.091***

a *** = *p*<0.001; ** = *p*<0.01 and; * = *p*<0.05.

**Figure 4:**
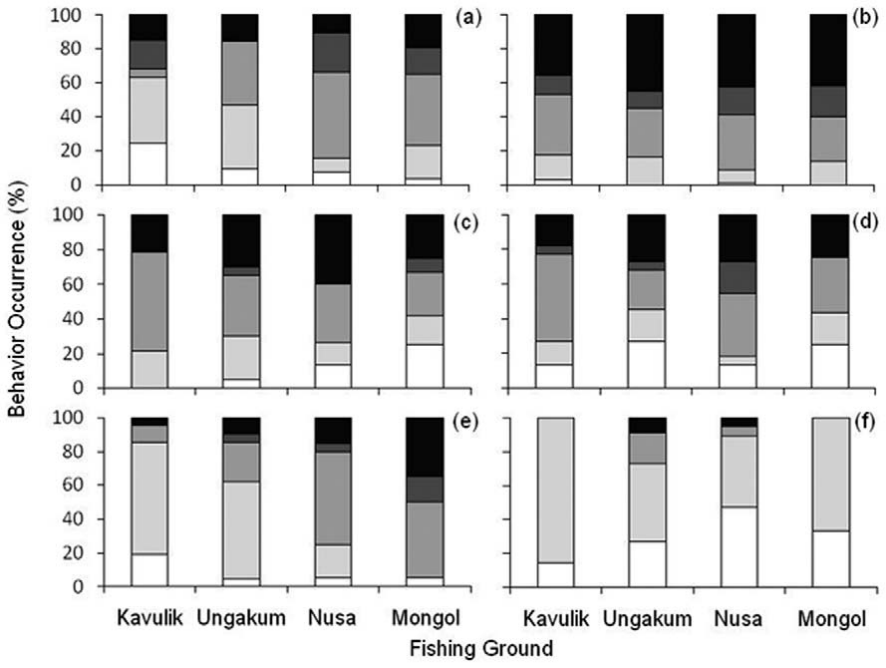
Pre-flight behavior of six reef fish families across four fishing grounds with different fishing pressures. Occurrence (%) of pre-flight behavioral categories in: (a) Acanthuridae; (b) Scaridae; (c) Balistidae; (d) Lutjanidae; (e) Mullidae; and (f) Serranidae across four reef areas in Papua New Guinea. Darkest shading indicates no notice behavior, followed by tacking away, orientating towards refuge, watching, and inspecting as shading becomes lighter.

Pre-flight behavior varied among families (Fig. 4), but only Acanthuridae and Mullidae showed changes in pre-flight behavior with increasing fishing pressure. Chi-squared tests indicated that least-wary behavior (“inspect/watch”) showed significant differences among areas for Acanthuridae (χ^2^ = 39.36, d.f. = 9, p<0.001). Within this family, focal fishes least-wary behaviors (“watch/inspect”) became less frequent as fishing pressure increased, while the more-wary behaviors (“orientation” and “tacking”) became more frequent (Fig. 4a). Mullidae showed a similar response to increased fishing pressure (χ^2^ = 39.55, d.f. = 9, p<0.001), with least-wary behavior decreasing as fishing increased (Fig. 4e). Although there was no significant difference in pre-flight behavior between fishing areas for Serranidae, this family exhibited less-wary behaviors, even at the highest fishing pressures (Fig. 4f).

Rugosity did not differ significantly between grounds (One-way ANOVA; F_(3, 36)_ = 1.74, p = 0.176), with a mean value across all areas of 2.83, indicating moderately complex reef systems in each area.

## Discussion

### Fishing pressure

Predator escape theory predicts that as intrinsic level of threat increases in an organism's surroundings, wariness (e.g., FID) will also increase [2]. This is supported by both experimental studies [41], [42] and field observations [12], [43]. We found that this prediction also holds true in the context of increasing human predation on coral reef fishes, although the behavioral response of fishes to increasing fishing pressure varied by family, and with target status. For example, Acanthuridae and Scaridae, which are the 1st and 3rd most commonly spear fished families in the region [39], showed the highest sensitivity to increased fishing pressure, while Lutjanidae and Serranidae, both of which are primarily caught by hook and line and more rarely caught by spear gun, showed no significant changes in FID between fishing pressures [16]. This concurs with FID estimates for *Lutjanus gibbus* in a previous study in PNG [12]. Serranidae, by contrast, showed a FID less than the effective range of spear guns at all sites. This lack of wariness may be due to the Serranidae being some of the predominant natural predators on coral reefs, and the low number of natural predators for this family [44], or due to territorial defense postures to perceived competitors [45].

We found little difference in FID between Kavulik NTA and the low fishing pressure area (Ungakum) across all families in this study, which could have several plausible explanations. First, these similarities could be explained by poaching occurring in the Kavulik NTA. However, the NTA is situated directly in front of the village, which facilitates monitoring [39], and community members report high compliance. Consequently poaching is an unlikely explanation for the similarities in FID between Kavulik and Ungakum. A second, alternative explanation could be due to low levels of fishing occurring at Ungakum. Both Ungakum and Kavulik are exposed to the north-west monsoon, which blows from November to April. During this time, fishers rarely venture beyond sheltered lagoonal waters (FAJ personal observation); fishing grounds at Ungakum may only be fished for six months of the year, and may not have been regularly fished prior to the study commencing due to unseasonal weather. Therefore, the impact of fishers within the Ungakum fishing area may not be high enough for wariness of fishes to be impacted, and subsequently FID to be affected. A third likely, but unconfirmed explanation is that similarities in FID between the two areas may be associated with the age of the Kavulik NTA (∼2 years at the time of this study) relative to age of the fish population. Prey fishes are able to gather information about the threat context in which they are present through both experience and social learning [21], and recall of predator attributes has been shown to occur after a gap of two years between encounters in minnows [46]. *Ctenochaetus striatus* individuals surveyed at Kavulik would be between 5 and 10 years of age [47], while the species of Scaridae surveyed are predicted to be from 3 to 5 years old [48]. Thus, the relatively recent no-take status at Kavulik means that fishes with previous experience of human predation, and consequently higher FID, were likely to still be present within its boundaries. At this point, it is not known how long fishes recall threats and adjust their FID accordingly. Future research into recall of appropriate flight response will be necessary to confirm this potential explanation.

The broad results from this study (that FID in fishes increased with fishing intensity) are consistent with previous research, but some details differ. In particular, Feary et al. [12] reported relatively greater FID within areas open to fishing for all target fishes than estimated in the present study (with the exception of the Acanthuridae). Likewise, estimates of Scaridae FID were markedly lower than either this study or by Feary et al. [12], both inside and outside a 26 year old NTA in Barbados [15]; the latter may be explained by low exploitation pressure in fished areas near the Barbados NTA [49] compared to fished areas in Papua New Guinea. However, differing methodologies may make direct comparisons between studies difficult. First, the methods of approach used in this study were designed to emulate a spear-fisher. These included descending away from the target fish and keeping flat and close to the substrate. These techniques may reduce the distance at which a fish becomes aware of the approaching observer and therefore initiates flight; such techniques were not used in either Gotanda et al. [15] or Feary et al. [12]. Second, both Feary et al. [12] and Gotanda et al. [15] used SCUBA to conduct FID surveys, although reports show that fishes can learn to associate the noises generated by SCUBA equipment, or the appearance of divers, with increased food availability [17], [50], and could learn to be wary of these noises where associated with spear fishing.

### Size

At no/low fishing pressures, size was not a factor explaining variation in FID, but at higher fishing pressures this factor became significant in explaining FID. The role of body size in determining FID in fishes remains poorly understood [19]. Optimal fitness theory predicts an increase in fishes' FID with increased body size, due to higher total investment relative to potential benefits (contributions to inclusive fitness), that may be gained by fleeing later [2], [51]. However, there is still conflicting evidence for the application of this theory to coral reef fishes. For example, body size in Caribbean parrotfish was the largest single determinant of increases in FID [15], while within Indo-Pacific reef fishes body size was unimportant in determining FID [12], and has been shown to be negatively correlated with reaction distance (not FID) to natural predators [52]. Here we have reported results that, while supporting the theoretical role of body size on FID, indicate that the relationship between body size and FID varies with fishing pressure.

The eco-morphology of predator/prey relationships should be taken into account when considering how body size may impact FID [53]. Smaller prey is more cryptic, harder to identify, and metabolically less profitable to target than larger sized prey [54]. These attributes are likely to reduce attractiveness of prey to predators, and result in lower prey FID [1]. As fishes grow larger, their locomotive ability grows, and their ability to avoid a predator increases, which potentially decreases FID [55]. Predator prey-size preference is also influential; fishes generally tend to consume prey whole [53], which places restrictions on the upper limit of prey size they can ingest. For example, a study on the reaction of a small coral reef fish (*Dascyllus trimaculatus*) to models of a predator, demonstrated that larger individuals were less wary [52], possibly because they are larger than can be handled by the size of predator.

The optimal size of prey for a predator is when prey body depth ∼0.6 gape width [54], although during a food deficit, predators may take larger prey [53]. Therefore, we hypothesize that FID will slowly increase with body size until body depth exceeds 0.6 gape width of the largest predator before: 1) remaining constant; or 2) decreasing as predation becomes less common due to increased handling time. Due to depletion of reef sharks [56], [57], predation escape via increased body size in coral reef fishes may be increasingly common, or may be occurring at lower prey body sizes. Given this assumption, we would not expect a significant impact of body size on FID in NTAs, a hypothesis supported by both this study and Feary et al. [12]. In fished areas however, humans may play a similar role to sharks by targeting larger fishes. Thus, FID would likely increase with body size, as reported here and in the Caribbean [15]. This may explain the non-significant impact of FID where fishes' exposure to fishing is low, but the increased impact when exposure to fishing is higher. In fact, spear fishers may preferentially target larger fishes due to increased body depth providing a greater target area. This may partially explain why the “taller” bodied Acanthuridae make up a large proportion of the spear-fish catch [39]. While body-depth may not be a limiting factor in human predation, there are other limits of handling capacity (e.g. power of spear gun, preference for fish size) that may afford a size refuge for fishes in fished areas, but most likely at larger body sizes than found for fishes surveyed in the present study.

There are alternative explanations for increasing FID with increased body size (discussed in Gotanda et al. [15]), including the importance of observer starting distance and increased visual acuity of prey fishes. Observer starting distance has been shown to be positively correlated with FID, as prey individuals are aware of predator focus earlier, and for longer [3]. As larger individuals are more easily identified from distance, compared to smaller prey, this may positively bias FID. In our study we controlled for this factor by standardizing starting distance across all fish sizes. Visual acuity of prey fishes may impact FID due to physiological changes with maturity, with visual acuity increasing with body size [58]. Similar to Gotanda et al. [15], we do not believe our results were impacted by differences in visual acuity between different sized fishes, due to all studies being accomplished in clear tropical waters and target fishes being close to or mature adults.

### Group size

Theoretically, as animals form larger groups both their field of view and total time spent scanning for predators increase [59]. This leads to higher alertness, identification of predators at greater distances, and a correspondingly increased FID [1]. However, within fishes increased group size tends to reduce FID [4], with risk dilution the primary benefit [60], [61]. Within the present study only Acanthuridae showed increasing FID with increasing group size. This response only occurred across, and not within areas (Table 2), and could indicate an independent anti-predation response to increased fishing pressure.

### Pre-flight behavior

This is the first study to examine pre-flight behavior in the context of increased fishing pressure. We demonstrated that pre-flight behavior varies by family, but that variance with fishing pressure is not universal, with both trophic group and life-history mediated responses. Lower trophic level families (i.e., Acanthuridae, Scaridae) displayed a higher proportion of wary behaviors (e.g., swimming away or immediate flight), while the highest trophic level family (Serranidae) showed almost exclusively less-wary behavior. The prevalence of immediate flight – the most wary behavior - in Scaridae may stem from fishes in this study generally being close to, or of terminal phase size, with corresponding higher reproductive value rewarding increased wariness [51]. In addition, while both the Acanthuridae and Mullidae showed the most obvious changes in behavior across fishing pressure, both families may have different vulnerabilities that drive change in behavior. Acanthuridae are one of the most heavily targeted families by spear fishers [39], and this status militates against non-wary behaviors being retained at even low fishing pressures. In comparison, Mullidae will rest on corals or rocks during the day, and in the Kavulik NTA one species, *Parupeneus crassilabris*, would often watch and not flee until the observer was within 100 cm, and would return to their perch within 30 seconds, often while the observer was still in the immediate area (FAJ, personal observation). This lack of wariness would make Mullidae an attractive target, despite being arguably a more difficult to target family due to relatively small body depth. Any reduction in the occurrence of this behavior, making them even more difficult to catch, is likely to have a large impact on frequency of targeting by spear fishers.

### Directions for future research

The basic prey model of optimal foraging theory predicts that a predator (i.e., in the present case a spear fisher) chooses prey based on profitability (potential energy gain per unit of handling time) [62]. This theory suggests that predators will concentrate on the most profitable prey, and as prey abundance decreases will switch to the next most profitable prey [53]. However, this assumes that all prey are equally vulnerable to capture, which is rarely the case, while profitability will change with consideration of prey attributes [63]. Theoretically, increases in FID in target fishes represent increasing difficulty of capture by spear fishers; therefore as FID increases, reducing the profitability of targeting a particular prey type, fishers will shift target preferences. As preferentially targeted families show higher FID, families with lower catchability due to smaller target areas (e.g. Mullidae) or greater intrinsic wariness (e.g., Mullidae or Lutjanidae) may play a greater role in the fishery; one speculative interpretation of our results may point to some preliminary support for this theory. FID for all but one family exceeded the effective range of spear-guns at the highest fishing pressure, while Mullidae and Balistidae FID only differed when the FID of Scaridae or Acanthuridae equaled or exceeded this distance (see Fig. 1). Whether this is due to prey switching by spear-fishers is unclear from our data, but presents an interesting avenue for future research. Currently, knowledge of how subsistence fishers prioritize which fishes they target is lacking. In order to better understand how changes in fish behavior may influence fisher behavior, factors that are important in fisher decision making, such as catchability, size, taste preference, cultural factors and ownership rights [64] will need to be explored more thoroughly. We have presented some interesting results that hint at prey switching by fishers due to fish behavior influencing catchability, and complement predictions that changing FID of fishes can influence the prey choice of fishers.

### Conclusions

Here we have presented the most comprehensive assessment to date of fishes' FID in relation to human predation. We have shown that fishes' FID varies with both fishing pressure and target status. Fishes' body size appears important in determining FID, however the relationship between size and FID of coral reef fishes is more complex than has previously been presented, and both prey and predator eco-morphology needs to be taken into account. While the data we present here indicates that pre-flight behavioral mechanisms may show promise in assessing fished status of some families of coral reef fishes, this behavior differs markedly across families and trophic groups. There may be scope to integrate FID into assessment of compliance and effectiveness of management of reef fisheries; however, variation in FID between species and geographic location requires local validation of FID prior to implementation as a successful management tool.
